# Postinfantile Giant Cell Hepatitis with Features of Acute Severe Autoimmune Hepatitis Probably Triggered by Diclofenac in a Patient with Primary Myelofibrosis

**DOI:** 10.1155/2018/9793868

**Published:** 2018-03-11

**Authors:** Pinelopi Arvaniti, Kalliopi Zachou, George K. Koukoulis, George N. Dalekos

**Affiliations:** ^1^Department of Medicine and Research Laboratory of Internal Medicine, School of Medicine, University of Thessaly, Larissa, Greece; ^2^Department of Pathology, Medical School, University of Thessaly, Larissa, Greece

## Abstract

Giant cell hepatitis (GCH) is commonly reported in neonatal and infantile liver diseases but rarely in adults where the term postinfantile GCH (PIGCH) is used. PIGCH is associated with many diseases, including drugs toxicity, viruses, and autoimmune liver diseases, with autoimmune hepatitis (AIH) being the most prevalent. We present a case of PIGCH in a 76-year-old female without known history of liver disease who suffered from an acute severe episode of hepatitis. After careful exclusion of other hepatitis causes by imaging, virological, immunological, and microbiological investigations, a diagnosis of acute severe AIH (AS-AIH) was established. The patient was started on corticosteroids but she did not respond and died 3 days later because of advanced acute liver failure. Postmortem liver biopsy showed typical PIGCH lesions. Physicians must keep this catastrophic entity in mind in cases of unexplained acute liver injury as, contrary to our case, prompt rescue therapy with corticosteroids may be life-saving.

## 1. Introduction

Syncytial giant cell hepatitis (GCH) is a condition characterized by inflammation and multinucleated hepatocytes, commonly found in wide spectrum of neonatal and infantile liver diseases [1]. On the contrary, this entity is considerably rare in adult patients with or without underlying liver diseases and, therefore, the term postinfantile GCH (PIGCH) is used for these cases. PIGCH is a nonspecific subtype of hepatitis which can be seen in a wide variety of inflammatory and cholestatic liver diseases, representing an enigmatic regenerative response of the hepatocytes to various noxious stimuli [2]. Neither pathogenesis nor the aetiology of PIGCH is known with certainty [2–5]. To date, only approximately 100 cases in adults have been described in the English literature during the past three decades [2]. PIGCH has been linked with drugs toxicity, viruses, and autoimmune liver diseases, with autoimmune hepatitis (AIH) being the most prevalent of all causes, accounting for approximately 40% of the published cases [2, 4–8]. The clinical outcome of patients depends on the underlying aetiology and varies from normalization of liver histology to progression to cirrhosis or even fulminant hepatitis [2, 6, 7].

Herein, we present a fatal case of histologically proven PIGCH with features of acute severe AIH (AS-AIH) probably triggered by diclofenac administration in a patient with myelofibrosis and no past history of liver disease.

## 2. Case Presentation

A 76-year-old woman was admitted to our department because of 10-day history of right upper quadrant abdominal pain that extended to the back and progressive deep jaundice. The patient had sought medical help 7 days before admission because of the same pain which was considered as of musculoskeletal origin and, therefore, nonsteroid anti-inflammatory drugs (NSAIDs) were prescribed by her doctor. The patient received diclofenac 50 mg PO twice per day, which was stopped at admission. Blood biochemistry at that time point was normal. However, there was no relief of the symptoms, while she mentioned the onset of nausea and vomiting accompanied by jaundice after 3 days of diclofenac administration but she continued taking the drug. The patient had a past medical history of primary myelofibrosis diagnosed two years ago and arterial hypertension under treatment with quinapril for fifteen years, while she was heterozygous for beta thalassemia trait. She denied ever consuming herbal agents and/or dietary supplements, intravenous or nasal illicit drugs, or alcohol use. Her past medical history for liver disease was also negative. Apart from jaundice, physical examination revealed hepatosplenomegaly with no signs of hepatic encephalopathy.

The laboratory work-up (abnormal values) was as follows: haemoglobin 10.8 g/dL, platelets 73 × 10^3^/*μ*L, international normalized ratio (INR) 2.32, albumin 3.3 g/dL, total bilirubin 21.6 mg/dL, direct bilirubin 15.4 mg/dL, AST 717 IU/L, ALT 679 IU/L, *γ*-GT 56 IU/L, and immunoglobulin G (IgG) 1760 mg/dL (upper limit of normal 1500 mg/dL). Chest X-ray showed pleural effusion on the left and the electrocardiogram right bundle branch block (RBBB). Arterial blood gases showed respiratory alkalosis (pH 7.52, pO_2_ 68 mmHg, pCO_2_ 28 mmHg, and HCO_3_ 22.9 mEq/L).

The remaining haematological, microbiological, virological, and biochemical parameters including blood cultures and investigation for hepatitis viruses A, B, C, and E as well as Epstein-Barr virus (EBV), cytomegalovirus (CMV), human immunodeficiency virus (HIV), herpes simplex virus (HSV), tuberculosis, leishmaniasis, brucellosis, and leptospirosis were negative. Liver autoimmune serology according to our standard protocols for the diagnosis of AIH was positive for antinuclear (ANA; titre: 1/80) and smooth muscle antibodies (SMA; titre: 1/320) by indirect immunofluorescence [9–11]; SMA specificity proved to be against F-actin antigen as confirmed by ELISA [9–11]. Abdominal ultrasonography revealed massive splenomegaly, which was attributed to primary myelofibrosis and hepatomegaly with dilation of splenic and portal veins. No signs of thrombosis were observed in hepatic, portal, and splenic veins.

Due to the presence of pleural effusion, in combination with RBBB and respiratory alkalosis in a patient with underlying procoagulant condition (primary myelofibrosis), a computerized tomography of pulmonary vessels was performed, which excluded the presence of pulmonary embolism/thrombosis. Liver biopsy at this moment although valuable was not performed because of absolute contraindications.

The differential diagnosis included all potential causes of acute severe hepatitis and, first of all, acute viral hepatitis, which was excluded by the careful negative serological and virological tests. Due to patient's medical history of primary myelofibrosis, acute Budd-Chiari syndrome and ischemic hepatitis as a consequence of massive pulmonary embolism or thrombosis were also considered. However, both diagnoses were excluded by imaging studies. In addition, alcoholic hepatitis was excluded as there was no history of alcohol consumption. The case of an idiosyncratic drug induced liver injury (DILI) was seriously taken into account in the differential diagnosis, because the patient had a history of NSAID use and especially diclofenac administration for the last 7 days before admission. Last but not least, AS-AIH was considered to be the most likely diagnosis, either genuine or induced by diclofenac administration (idiosyncratic DILI-induced AS-AIH), due to the exclusion of other causes and especially viral hepatitis, the presence of hypergammaglobulinemia, which is infrequent in DILI cases, and the positive liver autoimmune serology [9–11]. Indeed, the simplified score for the diagnosis of AIH without the histological data was 6 corresponding to the diagnosis of probable AIH [12]. It should be stated, however, that this score has not been developed for the diagnosis of acute severe cases of AIH as the typical or compatible histological lesions of chronic cases of AIH are typically completely different from those reported in AS-AIH [13, 14].

As the most probable diagnosis of the patient was AS-AIH (either pure AS-AIH or idiosyncratic DILI-induced AS-AIH), intravenous corticosteroids were initiated (1 g of methylprednisolone per day for 3 days followed by 1 mg/kg of prednisolone per day) as rescue therapy according to our experience and the guidelines of AIH management recently published by the European Association for the Study of the Liver (EASL) [13, 15]. Of note, multiple sets of urine and blood cultures before and after corticosteroid administration were sterile for bacteria and fungi infections, making the diagnosis of sepsis in a haemodynamically stable patient very unlikely.

**Figure 1:**
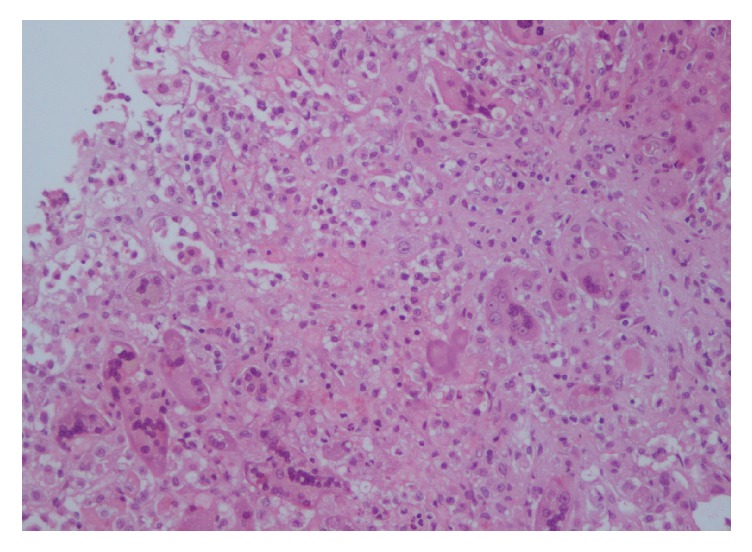
Postmortem liver biopsy showing confluent necrosis in a centrilobular area and syncytial multinucleated hepatocytes.

**Figure 2:**
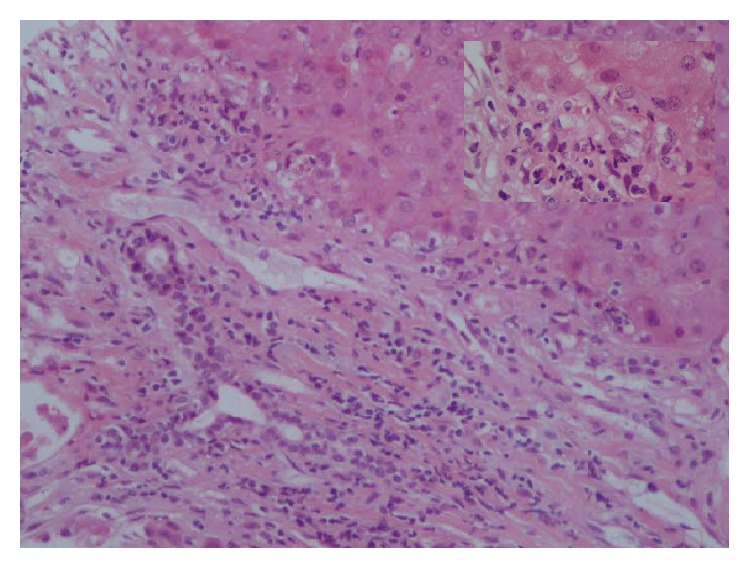
Postmortem liver biopsy showing portal tract with inflammation and focal minimal interface necroinflammatory activity (see also insert).

Despite the intravenous corticosteroid treatment, the patient deteriorated clinically, while her laboratory findings kept worsening (INR 4.74, albumin 2.66 mg/dL, total bilirubin 48.9 mg/dL, direct bilirubin 27 mg/dL, AST 1402 IU/L, and ALT 855 IU/L). On the 3rd day of hospitalization, she developed advanced hepatic encephalopathy and upper gastrointestinal hemorrhage, while her MELD score was 39. However, due to her medical history of primary myelofibrosis and her relatively advanced age, she was not considered as a good candidate for liver transplantation (LT). Finally, on the 4th day of hospitalization, she passed away. Her son did not consent to perform autopsy, but he consented to perform a postmortem liver biopsy, which revealed centrilobular confluent necrosis, focal interface hepatitis, portal inflammatory infiltration predominately with lymphocytes and portal fibrosis, cholestasis, and lobular inflammation with many giant hepatocytes (Figures 1 and 2). The whole histological picture was compatible with severe PIGCH probably as a result of AS-AIH [14].

## 3. Discussion

The following major point has been raised from the present case: although rare in adults, PIGCH must be kept in mind as a potential subtype of acute severe hepatitis in unexplained adult cases presenting with acute severe liver injury. Liver biopsy is a prerequisite for its diagnosis as characteristic morphological criteria are typically present in most patients including predominant presence of giant hepatocytes. A possible mechanism of their formation is the fusion of rosette-forming hepatocytes, although nuclear proliferation has also been proposed as part of the process, at least in a proportion of patients [2, 4, 5, 16]. Whether giant cells are formed by amitotic division or fusion of dividing cells remains debatable [17]. Nevertheless, syncytial GCH is considered as a very rare disorder in adults with an incidence of about 0.1 to 0.25% of all hepatic diseases [18], representing a nonspecific histological pattern with variable and rather obscure underlying aetiology [2, 4, 5].

From the aetiological point of view, PIGCH has been described during the course of several acute viral infections like hepatitis A, B, C, and E, EBV, HIV, CMV, and HHV-6 and a potentially unidentified paramyxo-like virus infection [2, 19–25]. In addition, many drugs such as methotrexate, 6-mercaptopurine, chlorpromazine, amoxicillin/clavulanate, doxycycline, NSAIDs, and herbal medicines have been reported as potential triggering factors for PIGCH development [2, 26–28]. Other conditions that have been implicated in the onset of PIGCH include autoimmune and/or immune mediated disorders such as systemic lupus erythematosus, autoimmune haemolytic anaemia, ulcerative colitis, and autoimmune cholestatic diseases, namely, primary biliary cholangitis (PBC) and primary sclerosing cholangitis (PSC), as well as haematological malignancies [2, 18, 29–35]. However, AIH is by far the most frequent disease associated with this rare histological entity in adults, actually accounting for almost 40% of all autoimmune related case series having features of AIH as in our case, one of few well-supported PIGCH cases [2, 8, 18, 23, 36–39]. Indeed, many patients with PIGCH described in case reports or case series have features of AIH as in our case, while one of the first well-documented PIGCH cases had positive ANA and SMA, marked hypergammaglobulinemia, and a good response to immunosuppressive therapy [2, 5, 7, 8, 18, 23, 36–39]. Of course the case of idiosyncratic DILI-induced PIGCH due to NSAID use cannot be excluded by certainty. However, the positive liver autoimmune serology, the presence of high IgG levels which is a distinct characteristic of AIH and not of idiosyncratic DILI [9–13], and the liver histology with features of AS-AIH even after corticosteroids pulse rescue therapy made the diagnosis of idiosyncratic DILI-induced PIGCH unlikely, although the histological distinction of DILI from AIH is sometimes very difficult due to significant overlap. Moreover, because of the rapid course of the disease, in this case, we were not able to distinguish between an idiosyncratic DILI-induced AS-AIH and a genuine (pure) AS-AIH, as the histological manifestations in these two entities may be indistinguishable [9, 11–14].

According to previous reports, giant cell formation is observed in more than two-thirds of the parenchyma and is most pronounced in zone 3. In some autoimmune related cases, giant cell transformation of the hepatocytes was seen in periseptal areas with multiacinar necrosis, increased inflammation, and some degree of periportal to severe fibrosis [2–6, 8]. Moreover, moderate to marked cholestasis has been described by others in relation, however, to AIH/PBC or AIH/PSC variant syndromes [17, 33, 34]. Under this context, the histological findings in our patient like centrilobular confluent necrosis, focal interface hepatitis, portal inflammatory infiltration predominately with lymphocytes, and lobular inflammation with many giant hepatocytes were in accordance with those described in previous reports in PIGCH patients with features of AIH, even though the possibility of modified liver histology because of high intravenous doses of corticosteroids should also be taken seriously into account.

The clinical course of PIGCH varies from acute liver failure to normalization of hepatic histology and progression to chronic hepatitis or even cirrhosis [2, 6, 40]. In general, the outcome depends on the underlying aetiology. However, the prognosis is often dismal with fatal outcome and survival rate of approximately 50% only if LT was not provided [2, 40]. As our case indicates, the high mortality rate is often due to the progression to acute liver failure, or even sepsis—though the latter was not confirmed in our case—in the setting of immunosuppression [2, 40, 41].

As PIGCH is a rare syndrome, there are no randomized controlled trials and, therefore, its treatment is not straightforward. However, corticosteroids and low-dose immunosuppressants like azathioprine have been used with success in some cases [37]. Aetiological treatment with pegylated interferon and ribavirin in patients with PIGCH related to hepatitis C has also been used, as well as with ribavirin alone for paramyxovirus-associated PIGCH [18, 42]. Nevertheless, despite treatment, the majority of patients will need LT due to the progression to cirrhosis or acute liver failure [2, 40, 41, 43]. Unfortunately, recurrent or de novo PIGCH has also been described after LT [43–46]. Accordingly, we used high doses of intravenous corticosteroids based on our experience and the recent EASL guidelines for AS-AIH management [13, 14] although with disappointing results.

In conclusion, PIGCH is a rare histological entity, which usually carries an unfavourable outcome. Its rarity makes proper therapeutic studies in large series of patients very difficult and, therefore, a proposal for potentially effective treatment cannot be made safely. However, when recognized after the exclusion of viral causes, a course of corticosteroids could be tried, since over 30% of the reported cases were attributed to AIH. Indeed, physicians must keep this catastrophic subtype of acute severe hepatitis in mind in cases of unexplained acute liver injury where prompt rescue therapy with corticosteroids may be life-saving.
